# Phase-tailored assembly and encoding of dissipative soliton molecules

**DOI:** 10.1038/s41377-023-01170-x

**Published:** 2023-05-17

**Authors:** Yusong Liu, Siyun Huang, Zilong Li, Haoguang Liu, Yixiang Sun, Ran Xia, Lisong Yan, Yiyang Luo, Huanhuan Liu, Gang Xu, Qizhen Sun, Xiahui Tang, Perry Ping Shum

**Affiliations:** 1 School of Optical and Electronic Information, Huazhong University of Science and Technology, Wuhan, China; 2 Key Laboratory of Optoelectronic Technology and Systems (Ministry of Education), Chongqing University, Chongqing, China; 3 School of Engineering and Materials Science, Queen Mary University of London, London, UK; 4 Department of Electronic and Electrical Engineering, Southern University of Science and Technology, Shenzhen, China

**Keywords:** Ultrafast lasers, Optical data storage

## Abstract

Self-assembly of particle-like dissipative solitons, in the presence of mutual interactions, emphasizes the vibrant concept of soliton molecules in varieties of laser resonators. Controllable manipulation of the molecular patterns, held by the degrees of freedom of internal motions, still remains challenging to explore more efficient and subtle tailoring approaches for the increasing demands. Here, we report a new phase-tailored quaternary encoding format based on the controllable internal assembly of dissipative soliton molecules. Artificial manipulation of the energy exchange of soliton-molecular elements stimulates the deterministic harnessing of the assemblies of internal dynamics. Self-assembled soliton molecules are tailored into four phase-defined regimes, thus constituting the phase-tailored quaternary encoding format. Such phase-tailored streams are endowed with great robustness and are resistant to significant timing jitter. All these results experimentally demonstrate the programmable phase tailoring and exemplify the application of the phase-tailored quaternary encoding, prospectively promoting high-capacity all-optical storage.

## Introduction

Streams of ultrashort light pulses delivered from mode-locked laser resonators stimulate potential applications in ultrafast science and information technology^1–6^. Here the elementary pulse entity, termed ‘dissipative solitons’ in contrast to the ‘pure solitons’ formed in conservative systems, originates from the dual interplays between the dispersion/nonlinearity and gain/loss^7–9^. Intriguingly, these ultrashort soliton entities can behave like particles and self-assemble into bound states of complex structures, endowed by the balanced interaction forces^10–13^. The parallel concept of ‘matter molecules’ is transferred into the multisoliton complexes, and many unveiled internal motions emphasize this light-matter analogy, which spreads the profound concept of dissipative soliton molecules (DSMs)^14^. Considering the increased complexity beyond the single entity, soliton molecules display more plentiful internal dynamics in the presence of energy exchange between each constituent, as well as would allow enhanced coding of optical-soliton bits^15–19^. Recent use of time-stretch dispersive Fourier transform (TS-DFT) based real-time spectral interferometry enables long-lasting studies on the transient multisoliton dynamics^1,17–19^. The degrees of freedom of molecular internal motions are anticipated by resolving the temporal separations and relative phases, in response to how far the molecular analogy reaches^20–26^. For applied stimuli, dynamic soliton molecules can support high-order encoding formats of all-optical information processing beyond the pulse-counting formats. This foresight of alternative encoding scheme is held by the precise switching between each bound state, arousing one striking question of programmable generation and control of molecular dynamics^20,27^.

Temporal optomechanical lattice induced trapping potentials enable an all-optical assembly of large soliton sequences in a photonics crystal fiber-based fiber laser resonator^5,28^. Soliton constituents can be globally ordered and locally spaced to form a supramolecular structure by tailoring long-range soliton interactions of different origins^10^. This panoramic scenario allows for the assemblies of soliton–soliton, soliton–molecule or molecule–molecule under controllable intra-molecular dynamics, with respect to the time scales ranging from tens of picoseconds to hundreds of picoseconds. While staring at the solitonic bound states down to several picoseconds even sub-picoseconds, precise manipulation of intra-molecular dynamics emerges into the scientific spotlight, echoing the degrees of freedom towards temporal separations and relative phases^29^. In consideration of the energy exchange within molecular structures, direct pump modulation is exploited to harness the multisoliton interactions for a rapid switching between discrete binding states, principally referring to the temporal separations^20,30^. Recent parallel work further performs on-demand harnessing of soliton molecules between each predetermined value of the temporal separation by varying the second-order group-velocity dispersion and dispersion losses^31,32^. Quaternary encoding and switching of soliton molecules are proposed beyond the binary format. These temporal-separation-switched intra-molecular manipulations would make these ‘potential applications’ realizable, as well as appealing to explore subtler assembly and control of soliton molecules, fueled by the degrees of freedom of internal dynamics, especially applicable to multiple encoding formats for all-optical storages.

The on-going discovery of internal molecular dynamics, via using the advanced spectral observations, in essence, can enrich the manipulation mechanism of soliton molecules^33–39^. Intrinsic insights into the mutual interactions reveal the scenarios of energy exchange within the binding structures, which emphasizes the inevitable routes for controlling the assembly of molecular patterns. Viewed from the real-time spectral interferometry, which is regarded as the ‘eyes’ in controllable laser systems, the fringe pattern is modified by external perturbations, generally indicating the beginnings of temporal-separation variations, synthesis and dissociation processes^10,40^. With regard to the superiority of the dynamic indicator and the applied foresights, the programmable tailoring of molecular phases is expected to be fuel debate; however, it is barely experimentally achievable for laser resonators. In this paper, we pursue a new quaternary encoding format based on the programmable tailoring of the internal dynamics of DSMs. Controllable manipulation on the energy exchange enables deterministic harnessing of soliton assemblies, accordingly developing four phase-defined regimes. By implementing electronic modulation of the gain supply, continuous switching among these regimes is realized, validating the reversibility and high fidelity of the programmable tailoring. In addition, the recorded real-time streams exemplify the availability of this phase-tailored encoding, which is highly desirable for multiple encoding and switching.

## Results

### System configuration and principle

**Fig. 1:**
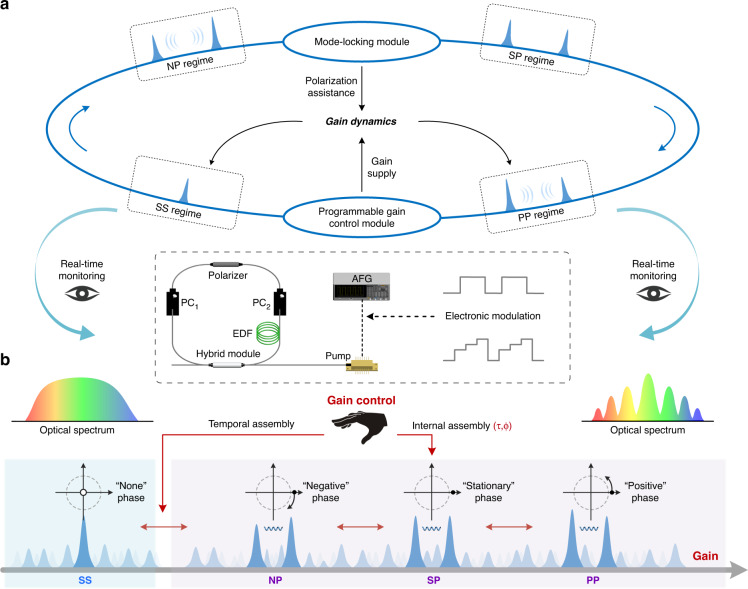
Schematic configuration and principle: artificial manipulation on temporal and internal assemblies of soliton molecules. **a** An ultrafast laser-based manipulation system, composed of a programmable gain control module, a mode-locking module and a real-time monitoring module. The experimental setup is shown in the inset (see more details in Materials and methods). The nonlinear polarization rotation (NPR) based mode-locking module enables polarization-assisted mode-locking. The cavity repetition rate of the fiber laser resonator is about 54.86 MHz. In addition, the operation wavelength is 1565 nm. DSMs with versatile internal motions are observed by adjusting the intracavity polarization and gain supply. The latter is modulated by electronic control signals, generated from an arbitrary function generator (AFG 31000, Tektronix) with a modulation bandwidth of 25 MHz. In addition, the real-time monitoring of the spectral evolution of dynamic soliton assembly is achieved by real-time spectral interferometry (see more details in Materials and methods). **b** Conceptual sketch of the artificial manipulation on the soliton assemblies from SS to NP, SP, and PP regimes, actuated by the increased intracavity gain supply. The evolving trajectories of the four phase-defined regimes indicate the ‘none’, ‘negative’, ‘stationary’, ‘positive’ molecular phases, respectively. The temporal assemblies (number of pulses) or internal assemblies (temporal separation *τ* and molecular phase *φ*) of DSMs can be deterministically harnessed by the precise gain control

To explore the controllable assemblies of DSMs, we develop an ultrafast laser-based manipulation system as shown in Fig. 1a. In addition to soliton singlets, plentiful multisoliton patterns can be supported by the fiber laser resonators under a sufficient gain supply^3,5,14^. Versatile internal motions are governed by the energy exchange between two adjacent constituents, which is important for phase-dominated soliton dynamics^11^. A real-time monitoring module is utilized to identify these particle-like behaviors by retrieving the temporal distributions and internal motions. Such particle-like behaviors can be harnessed by the tunable intracavity gain supply. High-speed electronic modulation of the gain supply is introduced to stimulate the artificial manipulation on the temporal assembly and internal assembly of DSMs (Fig. 1b). An appropriate increase in pump energy actuates the generation of a nascent soliton singlet from femtosecond fluctuations, while insufficient or superfluous pump energy usually leads to the soliton assemblies with unequal pulse intensities. The internal motions are connected with the pulse intensity relations between adjacent constituents. In particular, the molecular phase is a considerably sensitive indicator of external perturbations. While propagating in laser resonators, DSMs are manifested by different internal phase evolutions, which are defined into four regimes: soliton singlets (SSs), negative-phase (NP) soliton pairs, stationary-phase (SP) soliton pairs and positive-phase (PP) soliton pairs (Fig. 1b). On demand of the programmable tailoring of DSMs, gain modulation is introduced to deterministically harness the internal phase evolutions.

### Controllable assemblies of DSMs

**Fig. 2:**
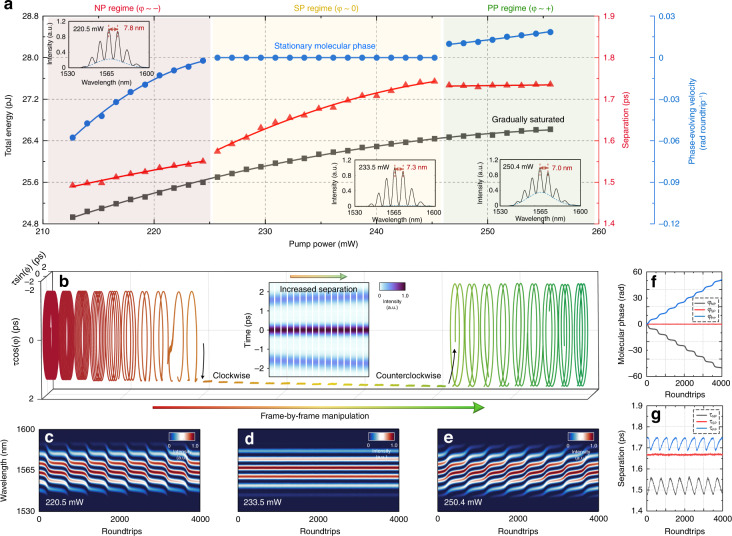
Phase-defined regimes of soliton assemblies. **a** Gradually saturated total energy (black curve), increasing temporal separation (red curve) and well-defined phase-evolving velocity (blue curve), all nonlinearly fitted. Frame-by-frame manipulation is implemented by a stepwise increase in pump power, from 212.7 mW to 255.6 mW, with a step of 1.3 mW. Two constituents are amplified by the increased gain supply, resulting in a saturated tendency of total energy. The soliton pairs are governed by the gain supply and defined into three regimes with respect to their different internal phase evolutions. Three representative averaged multishot optical spectra are shown in the insets, with the pump powers of 220.5 mW, 233.5 mW, and 250.4 mW, respectively. The free spectral ranges indicate the different temporal separations. **b** A 3D interaction space is introduced to show the frame-by-frame evolving trajectories of (*τ*, *φ*), especially the variation in phase-evolving velocity. Each frame represents 1000 roundtrips. The related frame-by-frame first-order autocorrelation traces in the SP regime are shown in the inset. **c**–**e** Successive spectral interferograms of the three soliton assemblies. Different drifting of spectral fringes implies intramolecular phase evolutions. **f** Three molecular phases. The different tendencies are regard as the symbols to identify the phase-defined regimes. **g** Three temporal separations. Each vibrating period corresponds to a 2π variation in the molecular phase

Tunable gain supply allows the deterministic assemblies with wide-ranging internal dynamics in the NP, SP and PP regimes. We initialize a typical NP soliton pair and make a frame-by-frame increase of pump power. The output power is transferred into the total energy of the dual-soliton assembly. The averaged evolving speed of the molecular phase, termed the “phase-evolving velocity”, is introduced to depict the sliding phase evolution. The variations of them in the frame-by-frame manipulation are resolved by real-time interferometry as shown in Fig. 2a. The adjustment of pump power can modify both the sliding phase evolution and the spectral modulation depth (see more details in Supplementary Fig. S1 and Section 1). The modification of these evolving molecular phases is depicted by the frame-by-frame trajectories of (*τ*, *φ*) (Fig. 2b).

For visualization, three phase-defined regimes of the DSMs are shown in Fig. 2c–g. The tendencies of the retrieved molecular phases (*φ*_*NP*_, *φ*_*SP*_, *φ*_*PP*_) indicate the different pulse intensity relations between the two constituents (Fig. 2f). In the NP regime, the trailing pulse is weaker than the leading one, which causes the different phase velocities of the two pulses in the gain medium and promotes the continuous negative phase accumulation^3^. The increased gain supply reduces the pulse intensity difference between the two constituents^3,11,13^. In the SP regime, the two constituents have the same pulse intensity. The temporal separation is enlarged with the increasing gain supply (see the inset in Fig. 2b). In the PP regime, the pulse intensity relation reverses and consequently yields the positive evolution in molecular phase. Periodic energy exchange within the soliton assemblies introduces the variation of pulse group velocity, resulting in the molecular vibration (*τ*_*NP*_, *τ*_*SP*_, *τ*_*PP*_) (Fig. 2g). In particular, soliton pairs can switch back and forth among the three phase-defined regimes through the precise control of the gain supply.

**Fig. 3:**
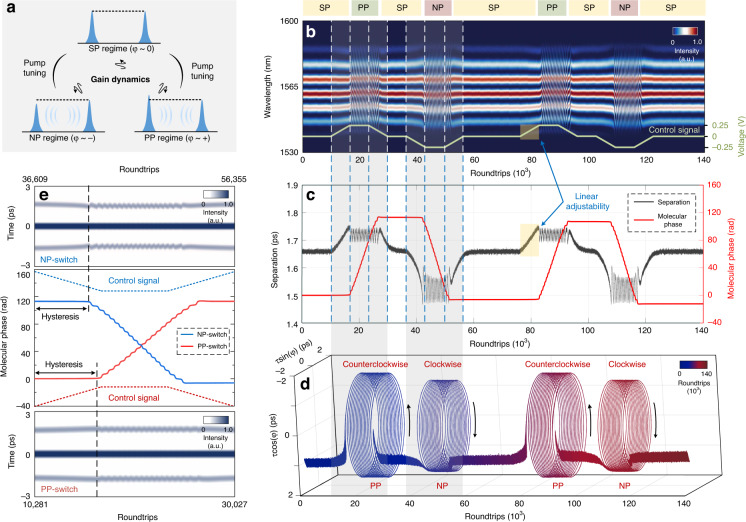
Continuous switching among the phase-defined regimes. **a** Schematic of the phase-defined soliton assemblies. The energy exchange within the DSMs is manipulated by the tunable gain supply, which enables continuous switching among the phase-defined regimes. **b** Successive spectral interferograms. The pump power is set at 236.8 mW and modulated by an electronic signal with a transfer efficiency of 52 mW V^−1^. The modulation period is 1.2 ms. Thus, the pump powers of the three deterministic soliton assemblies are chosen at 223.8 mW, 236.8 mW, and 249.8 mW, respectively. **c** Read-out of the temporal separation and the molecular phase during the entire harnessing process. The nearly linear variation in temporal separation (indicated by the yellow frame) verifies the linear adjustability. The rapid phase evolution is accompanied by the vibrating temporal separation. **d** A 3D interaction space for identifying the three phase-defined regimes, in line with the trajectories in Fig. 2b. **e** Consecutive first-order autocorrelation traces and extended views of NP-switching and PP-switching processes, with the related control signals indicated by the dotted lines

Sequentially, programmable electronic modulations of the gain supply are implemented to harness the phase-tailored assemblies of the DSMs. These internal phase dynamics are mainly affected by the gain dynamics, pulse intensity relations and energy exchanges between the two adjacent constituents (Fig. 3a). To illustrate the reversibility and fidelity of the programmable tailoring among the phase-defined regimes, we design a periodic electronic signal to modulate the pump power and actuate deterministic continuous switching (Fig. 3b). The successive spectral evolution of two harnessing periods is recorded. We tailor the soliton assemblies by combining the electronic control with phase measurement (Fig. 3c). Linear increase or decrease in pump power visualizes the switching between different phase-tailored soliton assemblies. The evolving trajectories in the 3D interaction space depict the variation of the phase-evolving velocity during the continuous switching, which can be used to identify the phase-defined regimes (Fig. 3d). The switching between different soliton assemblies is accompanied by hysteresis process (Fig. 3e). It can be optimized by using a rapid electronic modulation of the gain supply. More harnessing periods are recorded to validate the reversibility of the programmable tailoring (see these harnessing periods in Supplementary Fig. S2 and Section 2). In soliton molecule continuous switching, the fidelity is of considerable importance in multiple encoding and is quantified by calculating the Pearson correlation coefficient (see details in Supplementary Fig. S3 and Section 2). These highly reproducible phase-tailored soliton assemblies have potential applications in multiple encoding.

### Phase-tailored assemblies for quaternary encoding

**Fig. 4:**
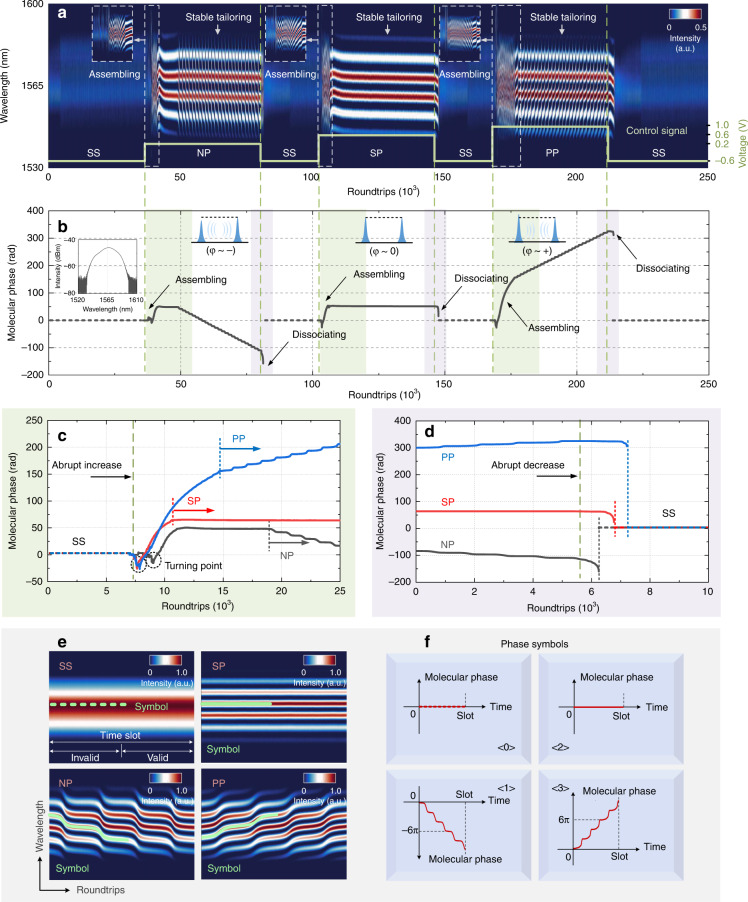
Phase-tailored assemblies and quaternary encoding principle. **a** Successive spectral evolution of continuous switching among the four phase-defined regimes. The pump power is set at 202.9 mW and modulated by an electronic signal embedded in the inset. The insets show magnified views of the assembly processes of the NP, SP, and PP soliton pairs, featured by rapid phase evolutions. **b** Retrieved evolving molecular phase. The SS regime is denoted by the dotted line. The four phase-defined regimes are easily distinguished. An optical spectrum of the SS is shown in the inset. The assembly and dissociation processes are harnessed with the gain modulation and are characterized in **c**, **d**. Abrupt increase or decrease in pump power stimulates the building-up or dying-out of an SS, but with different hysteresis processes. The turning points are probably related to the recoil voltage in the electronic modulation signal. **e**, **f** Recognizable symbols of the phase-defined regimes denote the spectral fringes (SS regime is denoted by the dotted line), constituting a new phase-tailored quaternary encoding format

The artificial manipulation of multisoliton patterns has attracted extensive interest in ultrafast optics^5^^,10,16,31^. Programmable tailoring of the molecular phase is implemented for multiple encoding. Beyond the pulse-counting-based binary encoding (see more details in Supplementary Fig. S4 and Section 3), we introduce these four phase-defined regimes (SS, NP, SP, and PP regimes) to represent the logical 0, 1, 2, and 3 of a new quaternary encoding format with high distinguishability. The four phase-tailored soliton assemblies can be prepared with the following pump settings: 171.7 mW (SS, encoded as ‘0’), 213.3 mW (NP soliton pairs, encoded as ‘1’), 234.1 mW (SP soliton pairs, encoded as ‘2’) and 254.9 mW (PP soliton pairs, encoded as ‘3’). To exemplify this phase-tailored quaternary encoding, we design an electronic modulation signal to stimulate continuous switching among these four phase-defined regimes. Successive spectral evolution of continuous switching among the four phase-defined regimes is recorded as shown in Fig. 4a. An abrupt increase in pump power induces the generation of a new SS from femtosecond fluctuations, while a decrease leads to the dying-out (Fig. 4b). The switching between the SS and the dual-soliton regimes involves the dynamic processes of the assembly and the dissociation. The assembly process generally follows four stages: raised relaxation oscillation stage, beating dynamics stage, transient bound state and stable soliton molecule^35^ (Fig. 4c). Different phase-tailored soliton assemblies will experience various hysteresis processes before reaching the deterministic regimes and are susceptible to external perturbations. The dissociation process is much faster than the assembly process for each phase-tailored soliton pair (Fig. 4d). The molecular phase is considered a sensitive indicator of external perturbations and can be used for the multiple encoding by implementing precise electronic modulation of the gain supply.

In practice, the key performances of the phase-tailored quaternary encoding are speed and fidelity. In the preparation of deterministic phase-tailored soliton assemblies, the main difficulty is how to maintain the same hysteresis processes. The direct electronic modulation can be upgraded to optimize the hysteresis process and the performance of the phase-tailored encoding. As a trade-off, in our tests, the 1-bit time slot is set as 200 μs (encoding speed ~5 kHz), including a 100-μs for valid data and another 100-μs for the hysteresis process (Fig. 4e, f). The spectral fringes are denoted by different symbols to distinguish the phase-defined regimes (Fig. 4e). The SS or SP regime is easily identified. In each time slot, we introduce the phase accumulation of [−∞, −6π] or [6π, +∞] to identify the NP or PP regime. Moreover, the all-fiber structure of the ultrafast laser-based manipulation system ensures the steady tailoring and transmission of the phase-tailored soliton assemblies. We perform a 30-min stability test for each phase-defined regime (see details in Supplementary Fig. S5 and Section 4) and develop a phase-tailored alphabet based on ASCII (see more details in Supplementary Fig. S6 and Section 5).

**Fig. 5:**
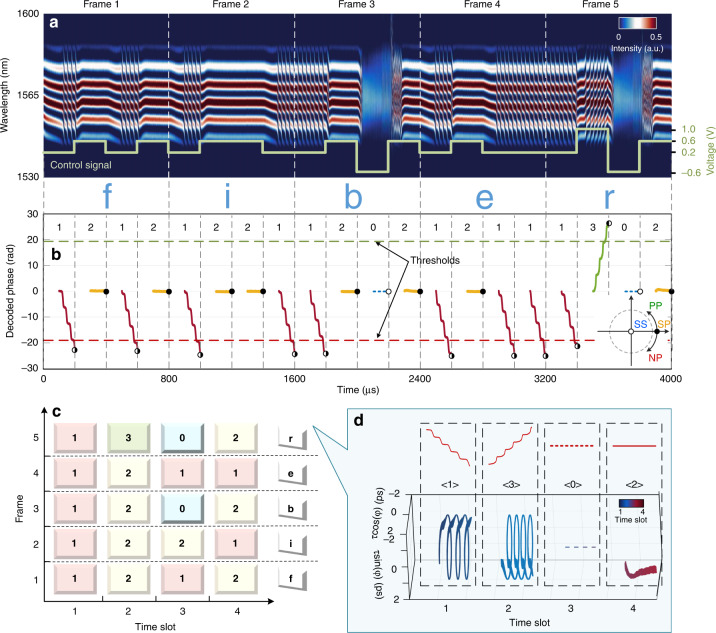
Exemplary multiletter encoding based on the quaternary format. **a** Successive recording of the encoded streams. The pump power is set at 202.9 mW and modulated by an electronic signal as embedded in the inset. Each frame is composed of four-time slots and represents a single letter. **b** Encoded streams are decoded as {1212|1221|1202|1211|1302}, representing a word {f|i|b|e|r}. The molecular phases of the four phase-defined regimes are with different colors and tendencies as depicted in the inset. The thresholds of −6π/6π are marked to identify the NP/PP regimes. **c** Visualization of each letter. An exemplary ‘r’ is portrayed in a 3D interaction space in **d**. The trajectory in each time slot agrees well with the corresponding phase-defined regime

To verify the availability of this phase-tailored quaternary encoding, an exemplary ‘fiber’ is encoded into real-time streams and decoded by retrieving the molecular phase (Fig. 5a, b). In each time slot, the valid information of the encoded molecular phase is retrieved while erasing the 100-μs hysteresis process. The two thresholds can be flexibly adjusted for the accurate identification of the phase-defined regimes. The recording of {1212|1221|1202|1211|1302} allows the construction of the encoded string {f|i|b|e|r} with high fidelity. For visualization, the multiletter streams are shown in Fig. 5c and an exemplary ‘r’ is depicted in a 3D interaction space (Fig. 5d). This multiletter test verifies the availability of phase-tailored quaternary encoding and the potential for a higher capacity in all-optical storage. The 1-bit time slot can be extended for more accurate identification (see more details in Supplementary Fig. S7 and Section 5). In the decoding process, the tendency of the internal phase evolution is the criterion to identify the phase-defined regimes. For long-haul transmission in fiber, to our knowledge, the chromatic dispersion and nonlinearity will affect the time-stretch process. Nevertheless, the tendency of the internal phase evolution can be still identified according to the different phase symbols. Thus, the phase-tailored encoding is also applicable to the long-haul transmission provided that the stretched pulses are not overlapped after the DFT. The phase-tailored streams are endowed with great robustness and possess high antijamming capability. The phase tailoring approach provides a new way to the artificial manipulation on the DSMs, which is desirable for the multiple encoding.

## Discussion

The controllable manipulation of the molecular patterns still remains challenging to explore more efficient and subtle tailoring approaches for satisfying the increasing demands. Apart from the temporal-separation-based harnessing of the molecular patterns, the molecular phase of DSMs is also expected to be harnessed. Recent studies of transient soliton dynamics provide strong supports for guiding the artificial manipulation on the molecular phase. Here, inspired by advanced applications of ultrafast optics in all-optical storage and optical computations, we develop an ultrafast laser-based manipulation system for the programmable tailoring of the molecular phase by implementing precise electronic modulations of the gain supply. The energy exchange within the molecular structures is manipulated to stimulate the deterministic harnessing of the phase-tailored soliton assemblies. Self-assembled soliton molecules are tailored into four phase-defined regimes, thus constituting the phase-tailored quaternary encoding format. Numerical simulations of the switching among different phase-defined regimes are conducted to confirm and interpret the physical mechanism of the programmable phase tailoring (see more details in Supplementary Fig. S8 and Section 6). Abrupt variations of the small signal gain *g*_0_ actuate the deterministic harnessing of the molecular phase of DSMs. The simulation results reveal the exact relations between the phase evolutions and the pulse intensities. In practice, the exemplary string {f|i|b|e|r} is presented to experimentally validate the availability of this phase-tailored quaternary encoding.

We remind that varieties of manipulation methods have been reported, which greatly enriches the knowledge towards the physical mechanism of molecular switching. The electronic modulation of the gain supply paves a direct way to harness the pulse intensities and facilitate the phase-tailored assembly of DSMs. For applied stimuli, the phase-tailored encoding with high fidelity is realized by implementing the programmable tailoring of the molecular phase into four regimes. The wide range of each phase-defined regime and the different phase symbols guarantee a low bit error rate (BER) and the accurate identification of the encoded formats. In particular, this direct electronic modulation can be improved by upgrading the electronic modulation signals to avoid the common recoil voltage and unstable voltage. The well-set voltages for these four phase-defined regimes can optimize the hysteresis of the continuous switching and the fidelity of the phase-tailored encoding. For increasing the speed of the quaternary encoding, faster phase-evolving velocities of the NP and PP regimes can be taken into account. Thus, we believe that the electronic modulation of the gain supply is considered as a direct and efficient way to realize the programmable tailoring of the molecular phase of the DSMs, and desirable for the applications in practical occasions.

For the moment, the topic of on-demand harnessing is currently driving considerable attentions. Based on the research thought that the transient dynamics guide the artificial manipulation on DSMs, we expect the lasting investigations by releasing the harnessing degrees of freedom. Particularly, more internal motions can be anticipated for enabling the artificial manipulation. The phase-evolving velocity can be quantified to encode information beyond the four phase-defined regimes. Moreover, the programmable tailoring of the temporal assemblies and the internal assemblies of DSMs allows for the higher-order encoding formats. While considering the molecular patterns with increased number of solitons such as the tri-soliton assembly and the quartic-soliton assembly^41^, the extended temporal-phase evolutions may accommodate more information for high-capacity all-optical storage. Naturally, the released degrees of freedom of the on-demand harnessing will bring about significant challenges for both the ‘eyes’ of the real-time characterization and the ‘hands’ of the precise manipulation. We consider two major avenues for the future investigations. One is to introduce the joint temporal-spectral analysis of the time-stretch dispersive Fourier transform and the time-lens measurements. The real-time full-field characterization can provide new insights into the internal motions, especially for the complex molecular patterns. ‘Hands’ can help control the laser parameters such as the gain, dissipation, dispersion, nonlinearity and polarization to drive the artificial manipulation. The released degrees of freedom of harnessing and the technical advances are expected to fuel this research topic and extend the application scenarios in the near future.

## Materials and methods

### Ultrafast fiber laser resonator

We investigate the programmable tailoring of the molecular phase of DSMs, which are generated from the ultrafast fiber laser resonator. The pump energy is programmed by implementing electronic modulations of a 980-nm semiconductor laser diode and is then injected into the laser resonator through a hybrid module, which is integrated by a wavelength division multiplexer (WDM), a 10:90 output optical coupler (OC) and an isolator (ISO). The emitted optical pulses are extracted from the 10% output port. The total length of laser resonator is 3.7 m, including a 0.8-m erbium-doped fiber (EDF, *β*_2_ = +0.061 ps^2^ m^−1^, EDF 80, OFS) as the gain medium and a 2.9-m single-mode fiber (SMF, *β*_2 _= −0.022 ps^2^ m^−1^). The cavity repetition rate of the fiber laser resonator is about 54.86 MHz. The NPR based mode-locking module is composed of a polarizer and two polarization controllers (PCs). The ultrafast fiber laser resonator with near-zero dispersion provides an abundant platform for investigating the phase-tailored soliton assemblies and the quaternary encoding.

### Real-time characterization of phase-tailored soliton assemblies

A 10-km SMF (*β*_2_ = −0.022 ps^2^ m^−1^) is used as a dispersive medium with a total dispersion of −220 ps^2^ for the time-stretch process. For soliton pairs with a temporal separation *τ*, the spectral modulation period 1/*τ* of the optical spectrum is mapped into the time domain and stretched to $\varDelta t=2\pi {\beta }_{2}L/\tau$. A 43-GHz photodiode detector (PD) and a 20-GHz high-speed oscilloscope (OSC, 80 Gs s^−1^, Teledyne LeCroy) are utilized to detect the successive spectral evolution of DSMs. And an optical spectral analyzer (OSA, YOKOGAWA) is used to monitor the optical spectrum for comparison. The first-order autocorrelation traces are calculated from the successive spectral interferograms by performing fast Fourier transform. The separation information and phase information (imaginary part) are extracted from the peaks of the first-order autocorrelation traces. However, the extracted phase information is in the range of [−π, π] and cannot represent the real internal phase evolution. The phase unwrapping is introduced to make the extracted phase information continuous by adding 2kπ. If the variation of two phases is over π rad, it is regarded as phase jump. The retrieved molecular phase should be consistent with the evolution of the spectral fringes in successive spectra interferograms. In particular, phase ambiguity is also important in the phase retrieval, especially during the switching between different soliton assemblies. The assembly and dissociation of soliton assemblies follow very complex dynamics and are represented by the rapid spectral evolution and unstable temporal separation, consequently resulting in the phase ambiguity^10^. Recent study has reported on machine learning strategies to reconstruct the multisoliton dynamics without the phase ambiguity, which have potential applications in ultrafast science^18^.

### Generation of electronic modulation signals

An AFG with a modulation bandwidth of 25 MHz is utilized to generate the electronic modulation signals. The emitted pump power is modulated by the electronic signal with a transfer efficiency of 52 mW V^−1^. Appropriate voltages related to the four phase-defined regimes are set to realize the high fidelity of the phase-tailored encoding. The direct electronic modulation can be upgraded to avoid the uncontrollable fluctuations of the gain supply, which is commonly induced by the recoil voltage and unstable voltage. Relying on the ASCII-based phase-tailored alphabet, arbitrary encoding can be realized by implementing the precise electronic modulation of the gain supply.

### Numerical simulations

To confirm and better interpret the mechanism of the phase-tailored soliton assemblies, numerical simulations are executed through a piecewise pulse propagation model in the scalar approach, which takes into account the different segments in the fiber laser resonator. The propagation model follows the extended nonlinear Schrödinger equation:

1 $$\begin{array}{l}\frac{\partial u(z,T)}{\partial z}=-\frac{i}{2}\left({\beta }_{2}+i\frac{g}{{\varOmega }_{g}^{2}}\right)\frac{{\partial }^{2}u(z,T)}{\partial {T}^{2}}\\\qquad\qquad\quad+\,i\gamma {|u(z,T)|}^{2}u(z,T)+\frac{g}{2}u(z,T)\end{array}$$

where $u(z,T)$ is the slowly varying electric field; *z*, *T*, *β*_2_, *γ* are the propagation coordinate, pulse duration, second-order dispersion parameter and nonlinear parameter, respectively; Ω_*g*_ is the gain bandwidth; *g* is the gain coefficient, modeled by $g={g}_{0}/(1+{P}_{ave}/{P}_{sat})$, where *g*_0_ is the small signal gain that is non-zero only for the EDF; *P*_*sat*_ is the gain saturation power; *P*_*ave*_ is the averaged power of pulses.

The saturable absorber in the fiber laser resonator is modeled by a transmission function: $T(I)=1-({a}_{0}/(1+I/{I}_{sat})+{a}_{ns})$, where *a*_0_, *I*, *I*_*sat*_, *a*_*ns*_ are the modulation depth, input intensity, saturation intensity and non-saturable absorbance, respectively. We emulate the experimental situation by adjusting the parameters of the fiber laser resonator. The internal phase evolution is retrieved from the successive spectral evolution.

## Supplementary information

Supplementary Information

## Data Availability

The data that support the plots within this paper and other findings of this study are available from the corresponding authors upon reasonable request.
