# Supplementary material for: Phase-tailored assembly and encoding of dissipative soliton molecules
Source: Light Sci Appl. 2023 May 17;12:123. doi: 10.1038/s41377-023-01170-x (PMC10192319; doi:10.1038/s41377-023-01170-x)
Supplement: Supplementary file 1 — Supplementary Information [file 41377_2023_1170_MOESM1_ESM.docx]

***Supplementary Information for***

**Phase-tailored assembly and encoding of dissipative soliton molecules**

Yusong Liu^1,2^, Siyun Huang^1,2^, Zilong Li^3^, Haoguang Liu^1^, Yixiang Sun^1^, Ran Xia^1^, Lisong Yan^1^, Yiyang Luo^2,*^, Huanhuan Liu^4^, Gang Xu^1^, Qizhen Sun^1,*^, Xiahui Tang^1^, Perry Ping Shum^4^

*^1^ School of Optical and Electronic Information, Huazhong University of Science and Technology, Wuhan 430074, China*

*^2^ Key Laboratory of Optoelectronic Technology and Systems (Ministry of Education), Chongqing University, Chongqing 400044, China*

*^3^ School of Engineering and Materials Science, Queen Mary University of London, London E1 4NS, United Kingdom*

*^4^ Department of Electronic and Electrical Engineering, Southern University of Science and Technology, Shenzhen 518055, China*

*^*^ Corresponding author*

Email: [yyluo@cqu.edu.cn](mailto:yyluo@cqu.edu.cn), [qzsun@mail.hust.edu.cn](mailto:qzsun@mail.hust.edu.cn)

**Contents**

**1.** Characterizations of optical spectra**2**

**2.** Reversibility and fidelity of continuous switching**2**

**3.** Gain-governed pulse-counting **4**

**4.** Stability tests for phase-tailored encoding**5**

**5.** ASCII-based quaternary encoding basis**6**

**6.** Numerical simulations**7**

**Section 1. Characterizations of optical spectra**

Limited by the scanning speed of a conventional optical spectral analyzer (OSA), particle-like behaviors of dissipative soliton molecules (DSMs) cannot be directly observed. In Fig. S1a, we show typical optical spectra of a stationary-phase (SP) soliton pair and a negative-phase (NP) soliton pair. The optical spectrum of the NP soliton pair is blurry and with a shallow spectral modulation, implying the rapid drift of spectral fringes. By increasing the pump power from 202.9 mW to 219.2 mW, the optical spectra of six NP soliton pairs are recorded as shown in Fig. S1b. Assisted with time-stretch dispersion Fourier transform (TS-DFT), these particle-like behaviors are resolved by retrieving the internal phase evolutions from the successive spectral evolutions (Fig. S1c). For the NP soliton pairs, the leading pulse is stronger than the trailing one, which causes the different phase velocities of the two pulses in the gain medium and promotes the continuous negative phase accumulation. And the increased gain supply reduces the pulse intensity difference between the two constituents and then modifies the phase-evolving velocity. The modulation depths of the optical spectra are seemingly related to the phase-evolving velocities of the NP soliton pairs. Both of them can be modified by the tunable gain supply.

In particular, for dynamic multisoliton molecules, their optical spectra are difficult to be recognized. We exhibit an optical spectrum of a dynamic tri-soliton assembly as shown in Fig. S1d, which is blurrier than the that of the dual-soliton assembly and implies the complicated internal phase evolutions. The two molecular phases (,) are retrieved as shown in Fig. S1e, both of them are with negative evolutions. The leading pulse should be stronger than the middle pulse and the trailing pulse. For the phase-tailored encoding, the tri-soliton assembly with composite internal motions can accommodate more information, which is desirable for supporting the higher-order encoding formats in all-optical storage.


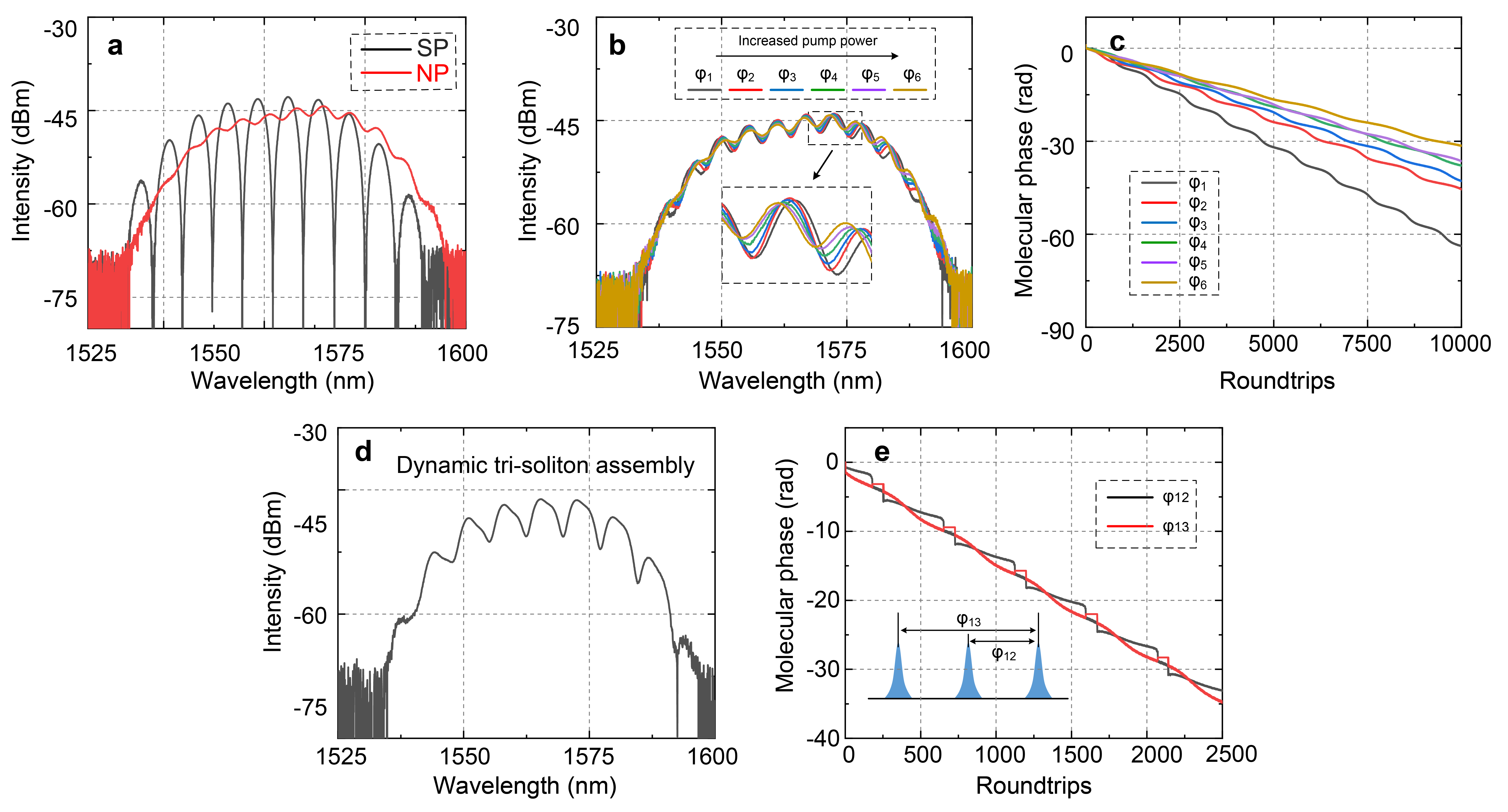


**Fig. S1. Characterizations of optical spectra.** **a** Exemplary optical spectra of an SP soliton pair and an NP soliton pair. **b** Optical spectra of the NP soliton pairs under the increasing pump power. **c** Retrieved internal phase evolutions of the six NP soliton pairs. **d** An exemplary optical spectrum of a dynamic tri-soliton assembly. **e** Retrieved molecular phases (,) of the dynamic tri-soliton assembly.

**Section 2. Reversibility and fidelity of continuous switching**

Beyond the two harnessing periods (see Fig. 3b in the main text), we also record over five harnessing periods with a decreased sampling rate of 5 GHz (Fig. S2a). The continuous switching between the 155000 and 195000 roundtrip is magnified as shown in Fig. S2b. The different spectral drifts agree well with the phase-defined regimes, validating the reversible operation of the soliton assemblies. However, limited by the sampling rate of 5 GHz, the spectral evolutions are blurry due to the low spectral resolution. In a 3D interaction space, the temporal separation is assigned to radius and the molecular phase is assigned to angle for better visualizing the evolving trajectory of ($\tau$,) to identify the three phase-defined regimes (Fig. S2c). This five-period recording validates the reversibility of the soliton molecule switching.


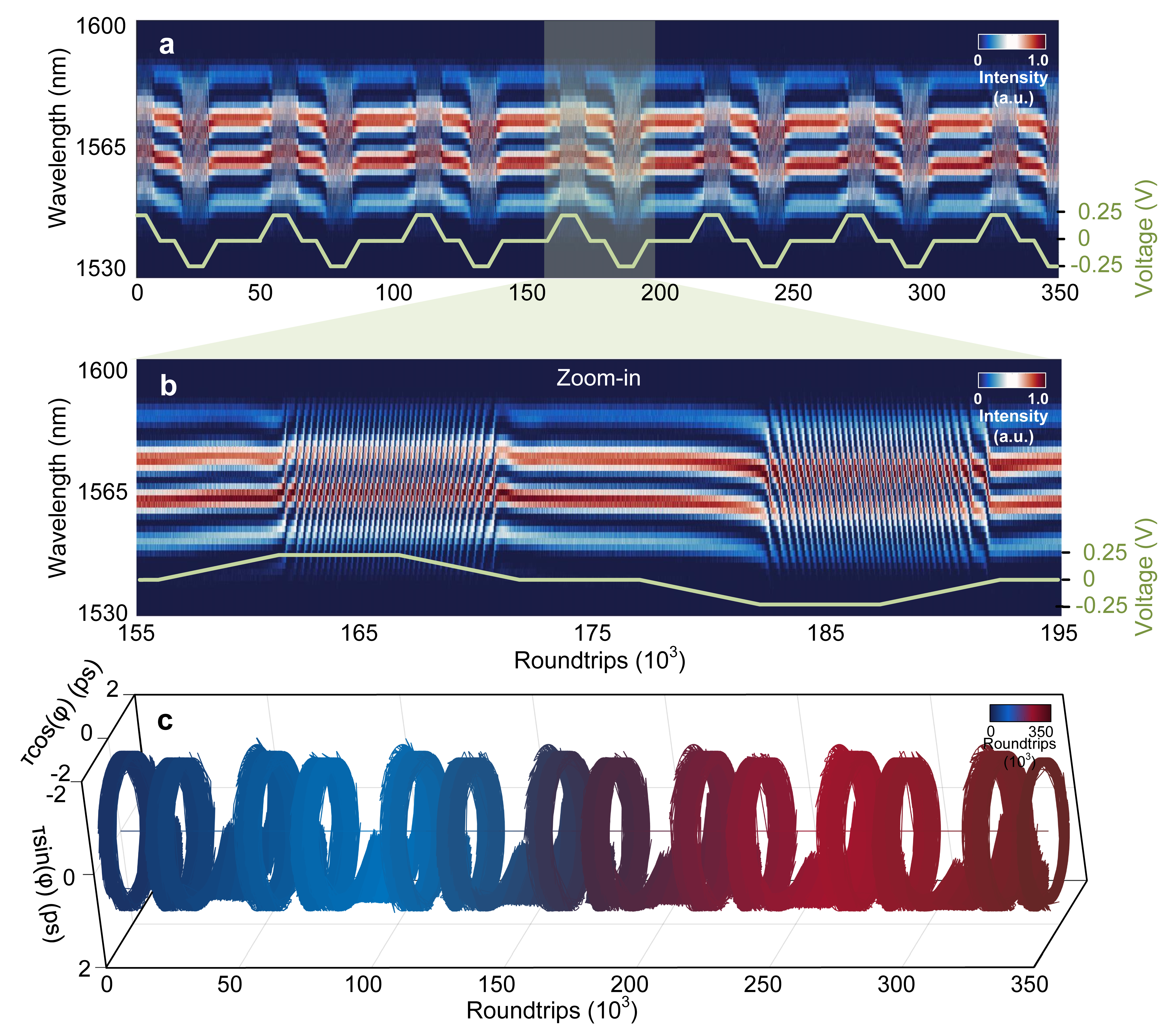


**Fig. S2. Continuous switching among the three dual-soliton regimes.** **a** Successive spectral interferograms of 350000 roundtrips, including over five harnessing periods. **b** One magnified harnessing period. **c** A 3D interaction space.

Moreover, quantifying the fidelity of the soliton molecule continuous switching is also of considerable importance in multiple encoding. Taking the five-period recording in Fig. S2a as an example, the first-order autocorrelation traces and the internal phase evolution are exhibited in Fig. S3a and b. In particular, Pearson correlation coefficient is introduced to quantify the fidelity of the continuous switching. It is calculated between the first autocorrelation traces in the first harnessing period and the following harnessing periods (Fig. S3c), verifying the high reproducibility and fidelity of the all-optical switching. In our work, the phase-tailored encoding with high fidelity is realized by implementing the programmable tailoring of the molecular phase of the DSMs. We also calculate the Pearson correlation coefficient of the internal phase evolution in each harnessing period (Fig. S3d). The calculated results further validate the high fidelity of the soliton molecule switching between the phase-defined regimes.


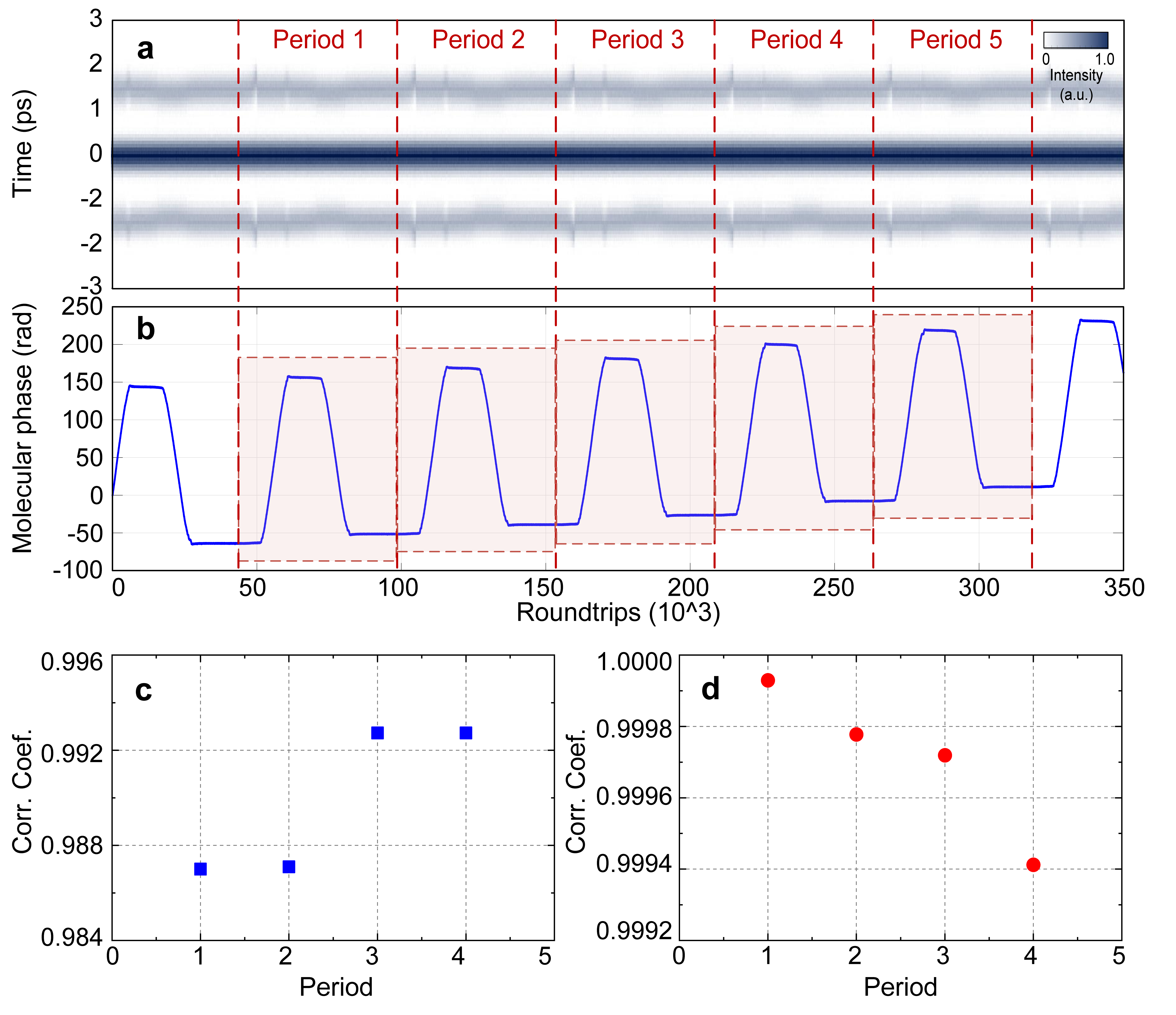


**Fig. S3. Quantifying the fidelity of the continuous switching.** **a** First-order autocorrelation traces of the Fig. S2a. **b** Read-out of the molecular phase, including over five harnessing periods. **c** Calculated Pearson correlation coefficient between the first period autocorrelation trace and the following period autocorrelation trace. **d** Calculated Pearson coefficient of the phase evolution between the first harnessing period and the following harnessing periods.

**Section 3. Gain-governed pulse-counting**

Apart from controllable all-optical switching among the three phase-defined dual-soliton regimes, a reproducible switching between a SS and a SP soliton pair is investigated as follows, which can be used as a binary pulse-counting format (Fig. S4). Two harnessing periods are displayed to verify the reversibility of the pulse-counting. An abrupt increase in pump power induces the generation of a new SS from femtosecond fluctuations, while a decrease leads to the dying-out. The switching between the SS and SP regimes involves the dynamic processes of the assembly and the dissociation. The assembly process generally follows four stages: raised relaxation oscillation stage, beating dynamics stage, transient bound state and stable soliton molecule. The dissociation process is much faster than the assembly process for each phase-tailored soliton pair. To optimize the assembly process, the direct electronic modulation should be upgraded to avoid the uncontrollable fluctuations of the gain supply, which is commonly induced by the recoil voltage and unstable voltage. The rise time and fall time of the electronic signals should be also taken into account. Additionally, the well-set voltages of the electronic modulations can also contribute to the steady control of the assembly process. Such deterministic harnessing paves an efficient path to tailor the number of dissipative solitons.


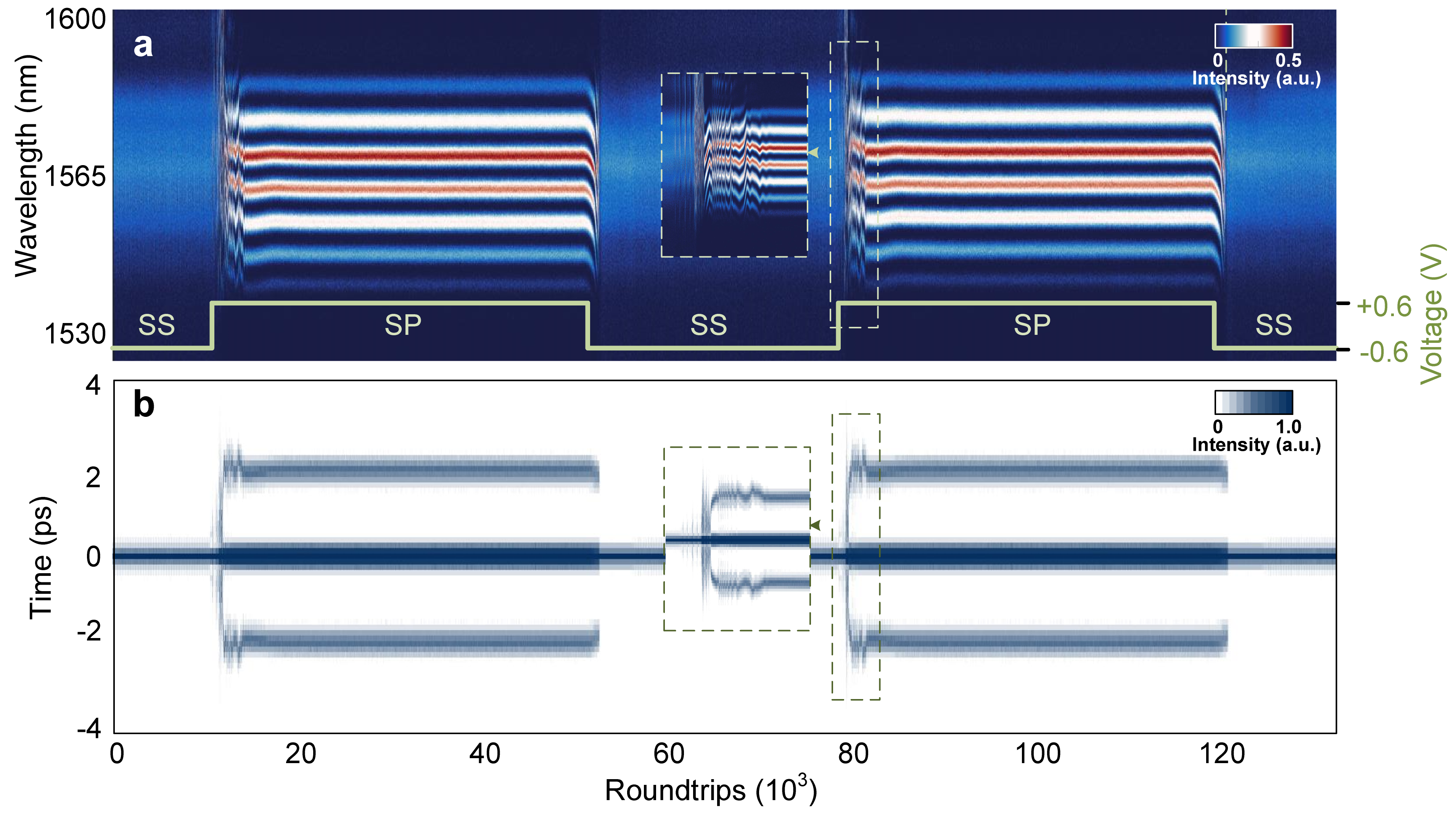


**Fig. S4. Gain-governed pulse-counting between the SSs and the SP soliton pairs.** The pump power is set at 186.6 mW and modulated by a square wave with an amplitude of 0.6 V and a duty cycle of 60%. **a, b** Successive spectral evolution and first-order autocorrelation traces of the pulse-counting. The close-ups of the assembly process are exhibited in the insets.

**Section 4. Stability tests for phase-tailored encoding**

In practice, the key performances of the phase-tailored quaternary encoding are the speed and fidelity. The steady tailoring of the molecular phase is also important for them. Here, we perform a 30-minite stability test for each phase-defined regime. The phase-evolving velocities of around -0.03 and +0.03 rad roundtrip^-1^ are chosen for the NP and positive-phase (PP) regimes. Different phase symbols of the phase-defined regimes are exhibited in Fig. S5, followed by the results of the stability tests. We characterize the variations of the pulse energy and center wavelength of the SS regime, as well as the temporal separations and phase-evolving velocities of the NP, SP and PP regimes. The stability tests for the NP and PP regimes validate the high fidelity of the programmable tailoring of the temporal separation and the molecular phase, while the SS and SP regimes confirm the stable center wavelength and the emitted energy. In the phase-tailored encoding, the tendency of internal phase evolution is the criterion to identify the phase-defined regimes. And the phase accumulation in each time slot is introduced to distinguish the NP or PP regime. Hence, the slight variations in phase-evolving velocities are tolerable (Fig. S5b and d). The 30-minite stability tests validate the steady tailoring of the molecular phase of the DSMs.


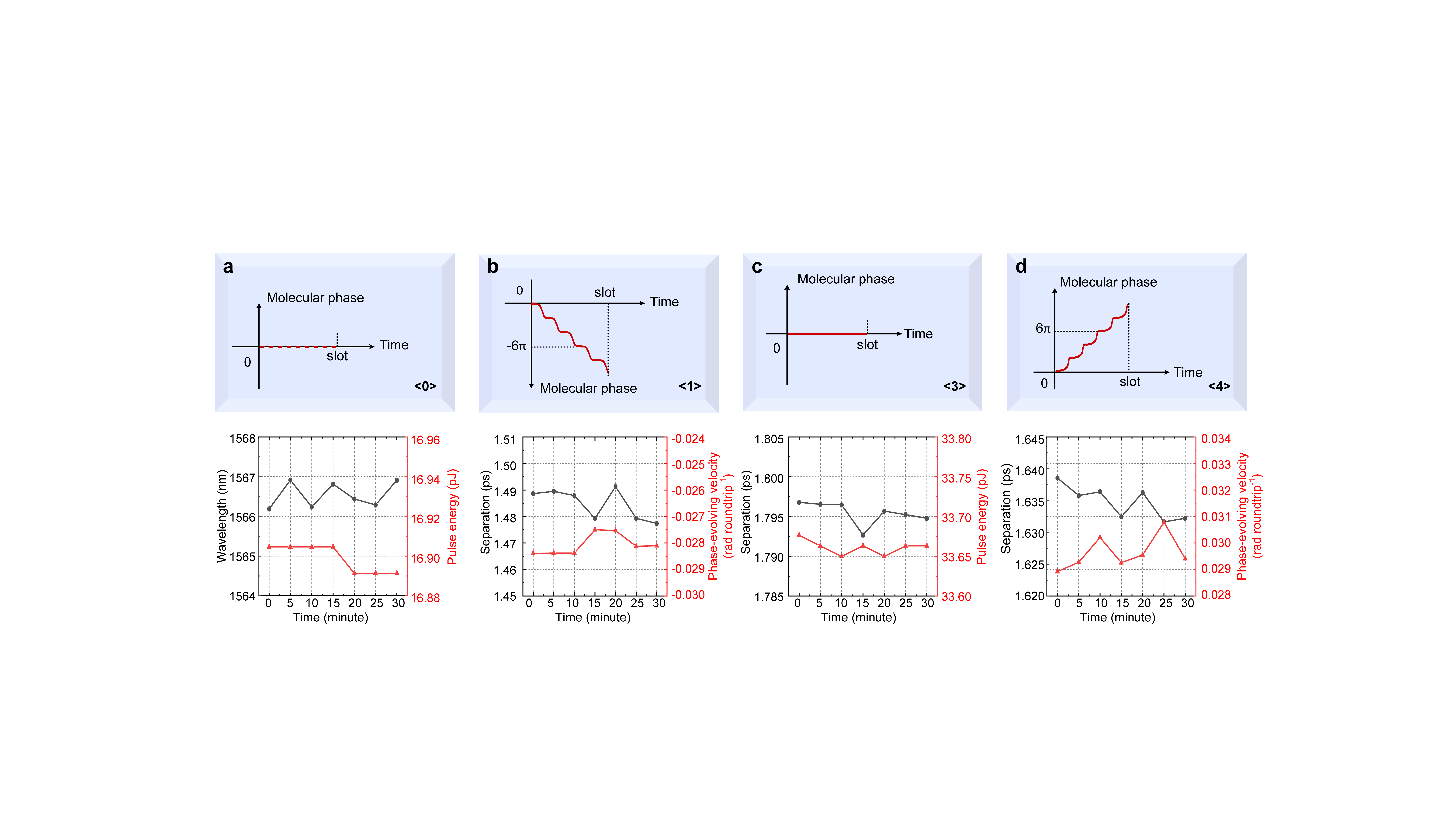


**Fig. S5. 30-min stability tests for the quaternary encoding format. a-d** Phase symbols and the related stability tests for the four phase-defined regimes.

**Section 5. ASCII-based quaternary encoding basis**

In the phase-tailored encoding, the information is encoded into the real-time streams and decoded by retrieving the internal phase evolutions from the successive optical spectra. To facilitate the phase-tailored encoding, we develop a table of a phase-tailored alphabet based on ASCII (Fig. S6). Each letter is composed of four time slots. The wide range of each phase-defined regime and the different phase symbols guarantee a low bit error rate (BER) and the accurate identification of the encoded formats.


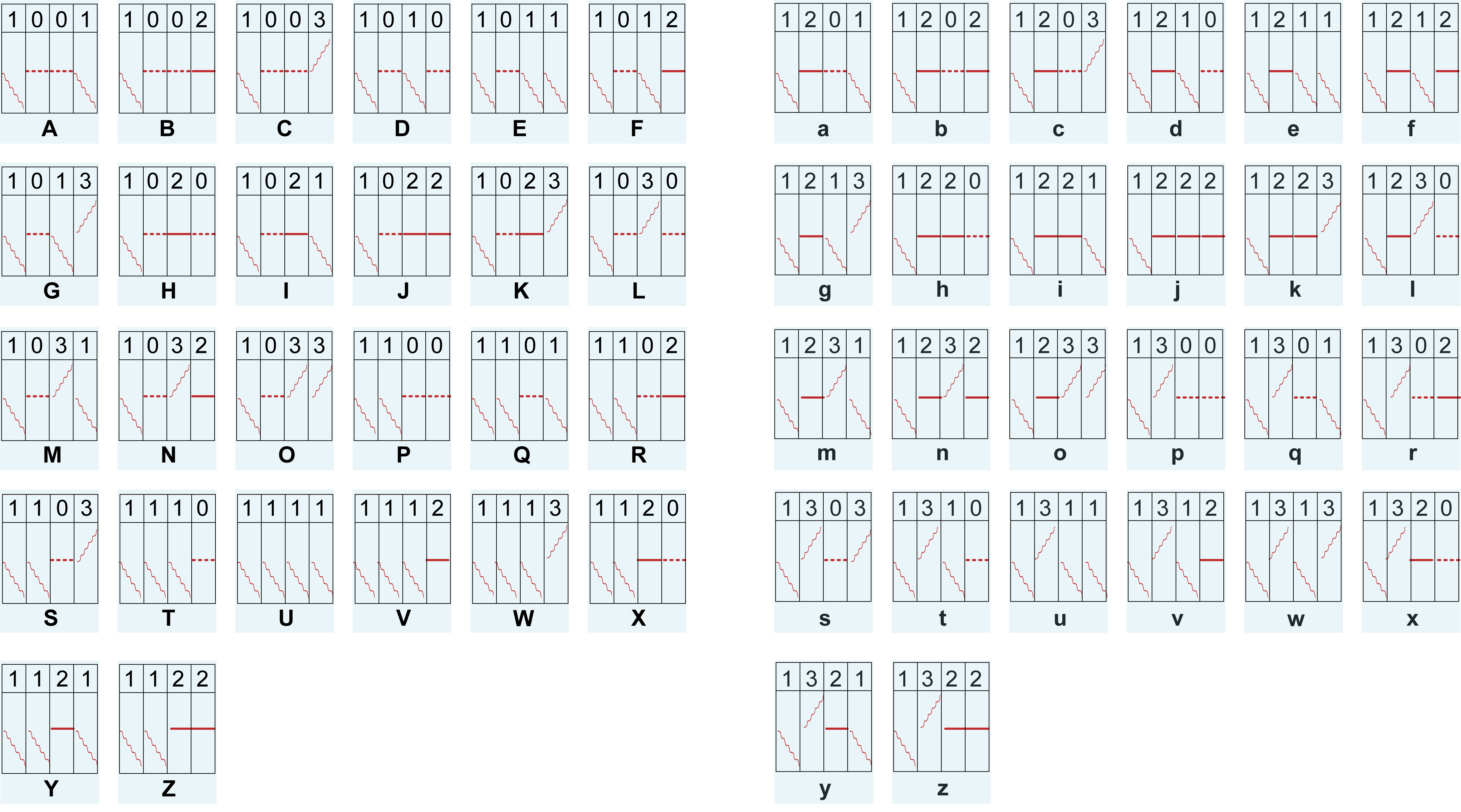


**Fig. S6.** A phase-tailored alphabet based on ASCII.

In our tests, the 1-bit time slot is set as 200 μs (encoding speed~5 kHz). However, while asking for more accurate identification of the phase-defined regimes, larger phase accumulation in each time slot is required. Here, the 1-bit time slot is extended to 400 μs (encoding speed~2.5 kHz), including a 200-μs for valid data and another 200-μs for the hysteresis process. We exemplify two multiletter encoding tests as shown in Fig. S7. The last names of the two authors ‘*liu*’ and ‘*huang*’ are encoded into real-time streams and decoded by retrieving the molecular phases. In each time slot, the valid information of the encoded molecular phase is retrieved while erasing the 200-μs hysteresis process. Compared to the results in Fig. 5, the phase accumulation in each time slot here is nearly doubled, consequently enabling the higher accuracy of the identification of the phase defined regimes. However, considering the key performance of the encoding speed, the 1-bit time slot of 200 μs is finally chosen for the phase-tailored quaternary encoding.


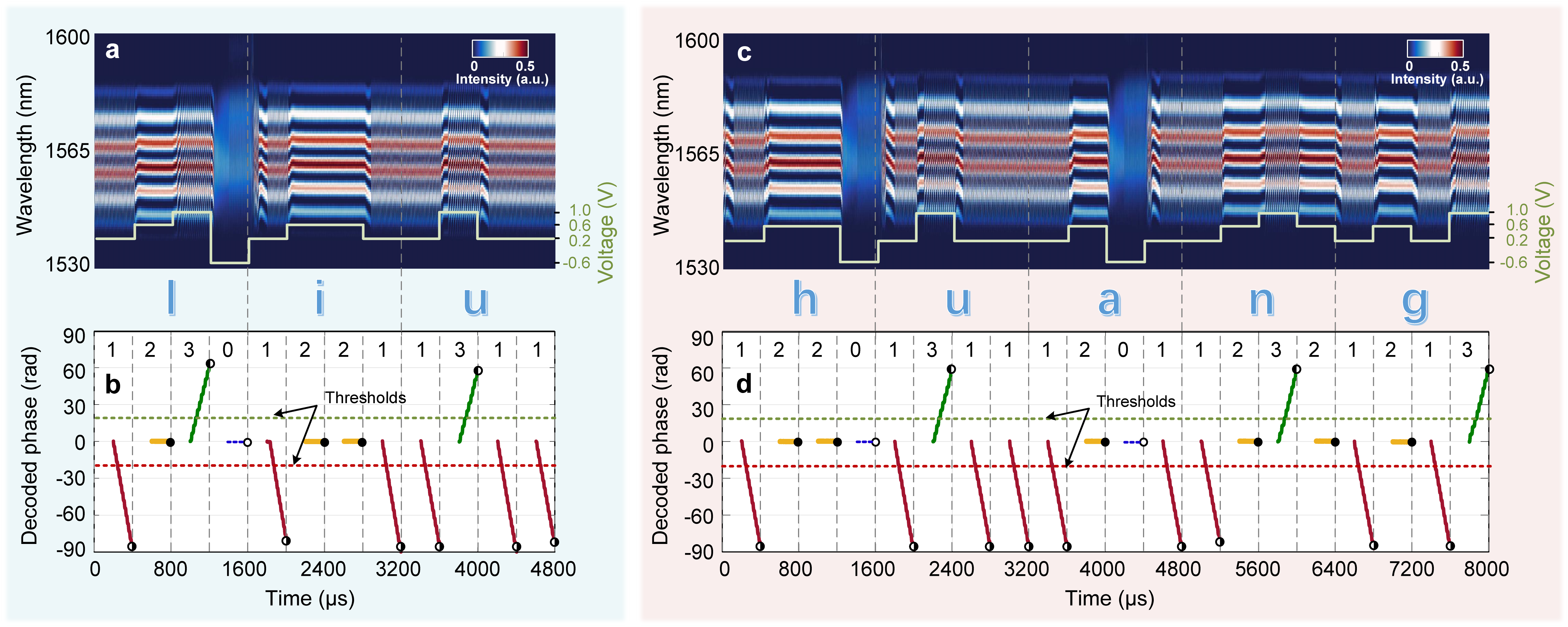


**Fig. S7. Exemplary multi-letter tests of ‘liu’ and ‘huang’ with a time slot of 400 μs.** The pump power is set at 202.9 mW and modulated by electronic signals with a transfer efficiency of 52 mW V^-1^. **a,b** Successive recording and resolving of the encoded string {l|i|u}, which is decoded as {1230|1221|1311}. **c**, **d** Successive recording and resolving of the encoded string {h|u|a|n|g}, which is decoded as {1220|1311|1201|1232|1213}.

**Section 6. Numerical simulations**

In our experiments, electronic modulations of the gain supply stimulate the DSMs to switch among the phase-defined regimes. To support and better interpret the physical mechanism of the switching dynamics, we have made numerical simulations of the switching between the NP, SP and PP regimes. Here, by setting the small signal gain $g_{0}$ at 35.08, we initialize a SP soliton assembly. An abrupt decrease of $g_{0}$ to 35.00 stimulates the SP soliton assembly to switch to the NP regime (Fig. S8a). The negative evolution of molecular phase and the pulse intensities of the two constituents are shown in Fig. S8b and c, verifying that the leading pulse is stronger than the trailing pulse in the NP regime. The oscillatory phase is ascribed to the oscillation of the pulse intensities of the two constituents. When the pulse intensities are equal, the phase difference remains constant, leading to the turning points of the molecular phase. The simulated results reveal the exact relations between the phase evolution and the pulse intensities.

Likewise, we initialize the same SP soliton assembly with $g_{0}$=35.08. An abrupt increase of $g_{0}$ to 36.00 actuates the switching towards the PP soliton assembly (Fig. S8d). The pulse intensity relation reverses (the trailing pulse is stronger than the leading pulse), consequently yielding the positive evolution in molecular phase (Fig. S8e). The stable PP evolution is accompanied with the periodic oscillation of the pulse intensities as shown in Fig. S8f. The numerical simulations provide the theoretical supports on the programmable phase-tailoring of molecular phase and improve the systematicness of our work.


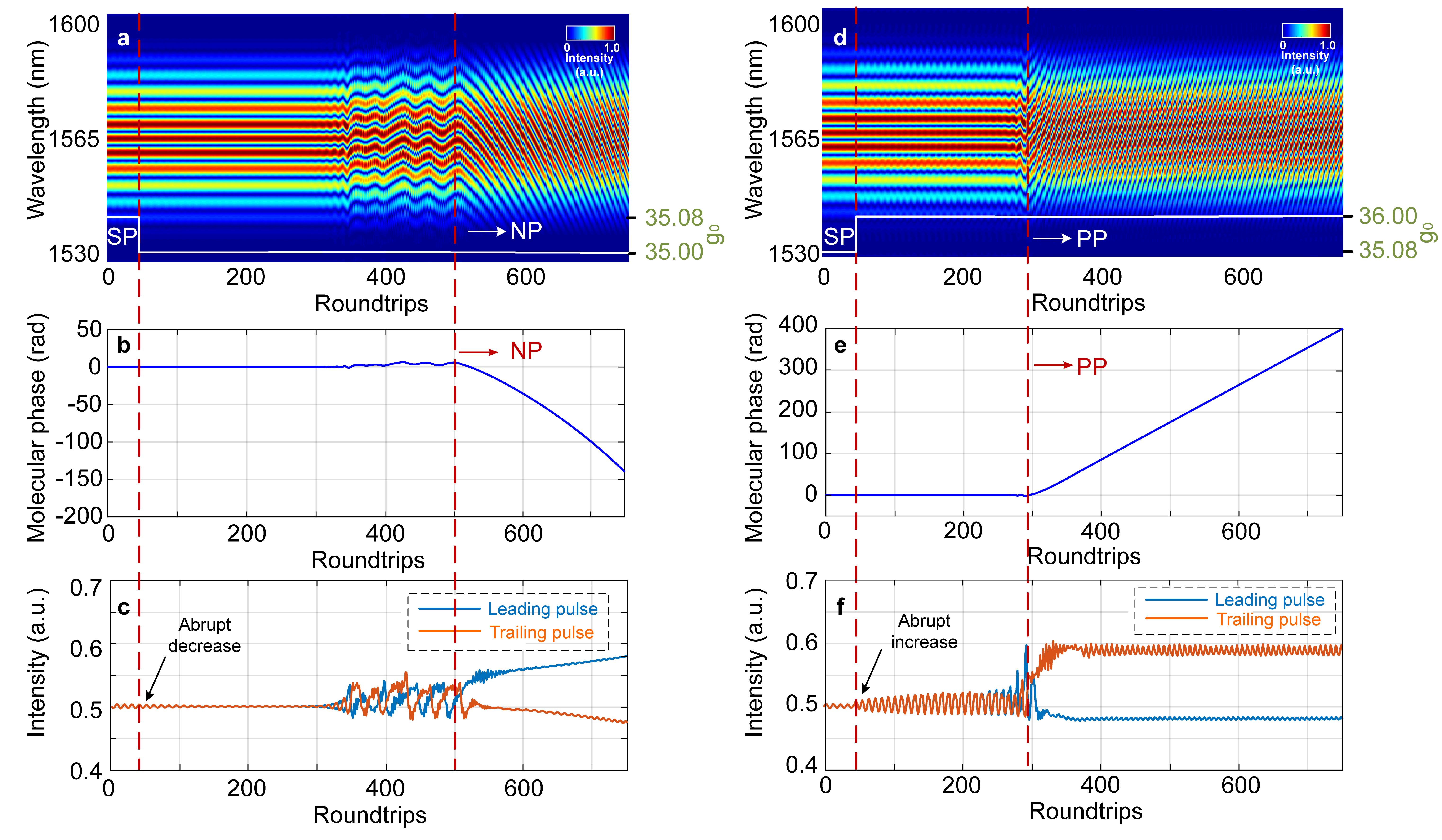


**Fig. S8. Numerical simulations of the soliton molecule switching.** Switching between the SP and NP regime: **a** Successive spectral interferograms. **b** Read-out of the molecular phase. **c** Pulse intensities of the two constituents. Switching between the SP and PP regime: **d** Successive spectral interferograms. **e** Read-out of the molecular phase. **f** Pulse intensities of the two constituents.
